# Evaluation of a new semi-automated Hydragel 11 von Willebrand factor multimers assay kit for routine use

**DOI:** 10.5937/jomb0-26008

**Published:** 2021-03-12

**Authors:** Marika Pikta, Timea Szanto, Margus Viigimaa, Sandra Lejniece, Dārta Balode, Kadri Saks, Valdas Banys

**Affiliations:** 1 North Estonia Medical Centre, Department of Laboratory Medicine, Tallinn, Estonia; 2 Tallinn University of Technology, Department of Health Technologies, Tallinn, Estonia; 3 Helsinki University Hospital Comprehensive Cancer Centre and University of Helsinki, Coagulation Disorders Unit, Department of Hematology, Helsinki, Finland; 4 North Estonia Medical Centre, Centre of Cardiology, Tallinn, Estonia; 5 Riga Stradins University, Riga, Latvia; 6 Riga East University Hospital, Chemotherapy and Hematology Clinic, Riga, Latvia; 7 Tallinn Children`s Hospital, Hematology Department, Tallinn, Estonia; 8 Vilnius University, Faculty of Medicine, Institute of Biomedical Sciences, Department of Physiology, Biochemistry, Microbiology and Laboratory Medicine, Vilnius, Lithuania

**Keywords:** electrophoresis, multimers, von Willebrand factor, von Willebrand disease, elektroforeza, multimeri, Fon Vilebrandov faktor, Fon Vilebrandova bolest

## Abstract

**Background:**

Accurate diagnosis and classification of von Willebrand disease (VWD) are essential for optimal management. The von Willebrand factor multimers analysis (VWF:MM) is an integral part of the diagnostic process in the phenotypic classification, especially in discrepant cases. The aim of this study was to evaluate the performance of a new Hydragel 11VWF multimer assay (H11VW).

**Methods:**

Analytical performance characteristics such as repeatability (intra-assay variability, in gel between track variation), reproducibility (inter-assay variability, between gel variation), sensitivity, EQA performance and differences between two commercially available VWF:MM kits (H5VW and H11VW) were analysed in healthy volunteers' plasmas using in-house prepared reference plasma.

**Results:**

Repeatability and reproducibility results of H11VW demonstrated acceptable and equivalent performance with previously verified H5VW. Participation in EQA was successful. No statistically significant difference was detected between H5VW and H11VW kits for different fractions of multimers: LMWM p=0.807; IMWM p=0.183; HMWM p=0.774.

**Conclusions:**

H11VW demonstrated acceptable analytical performance characteristics. H11VW kit conveniently offers a more significant number of samples on a single gel. H5VW and H11VW kits can be used in daily practice interchangeably.

## Abstract (sr)

**Uvod:**

Tačna dijagnoza i klasifikacija Fon Vilebrandove bolesti (VVD) su neophodni za optimalni rad. Multimerska analiza Fon Vilebrandovog faktora (VVF:MM) je u fenotipskoj klasifikaciji, posebno u slučajevima neslaganja, sastavni deo dijagnostičkog procesa. Cilj ove studije je bio da proceni performanse novog Hidragel 11VVF multimer testa (H11VV).

**Metode:**

Karakteristike analitičkih performansi, kao što su ponovljivost (varijabilnost unutar testa, u gelu između varijacije traga), obnovljivost (varijabilnost među testovima, između varijacije gela), osetljivost, EQA performanse i razlike između dva komercijalno dostupna VVF:MM seta (H5VV i H11VV) su analizirane na plazmama zdravih dobrovoljaca i interno pripremljenoj referentnoj plazmi.

**Rezultati:**

Rezultati ponovljivosti i obnovljivosti H11VV su pokazali prihvatljive i ekvivalentne performanse sa prethodno verifikovanim H5VV. Učešće u EQA je bilo uspešno. Nije utvrđena statistički značajna razlika između H5VV i H11VV setova za različite frakcije multimera: LMVM p = 0,807; IMVM p = 0,183; HMVM p = 0,774.

**Zaključak:**

H11VV je pokazao prihvatljive karakteristike analitičkih performansi. Korisno je što H11VV komplet nudi veći broj uzoraka na jednom gelu. Kompleti H5VV i H11VV se mogu koristiti naizmenično u svakodnevnoj praksi.

## Introduction

Deficiency or/and abnormality of von Willebrand factor (VWF) leads to von Willebrand disease (VWD), which is the most common inherited bleeding disorder [1]. Bleeding features are commonly characterised by mucocutaneous hemorrhage (e.g. epistaxis, menorrhagia), but hematomas and hemarthrosis may also occur in severe forms. The diagnosis of VWD presents many challenges: 1) there is a great overlap of clinical phenotypes and laboratory parameters between healthy individuals and those with type 1 VWD, and 2) a variety of increasingly specific laboratory tests are necessary for an accurate diagnosis of VWD [1]. The choice of the validated test panel is essential for the correct typing (type 1, 2 and 3 VWD) and subtyping of type 2 VWD. Due to the steadily increasing interest of VWD reclassification of prediagnosed VWD, the number of publications regarding VWD diagnosis has increased in recent years [2] [3]. In addition to first-line tests, such as factor VIII, VWF antigen and VWF activity assays, assessment of VWF multimers testing (VWF:MM) is important for the correct classification of VWD subtypes [1] [4]. However, the availability of VWF:MM is limited due to technical difficulties, variable results and long turnaround time of conventional VWF multimer techniques [5] [6] [7].

A novel semi-automated Hydragel 5VWF multimers assay kit (H5VW) has been already evaluated for use on the Hydrasys 2 Scan instrument (Sebia, Lisses, France) by several authors [8] [9] [10] [11] [12]. In May 2019, the VWF:MM analysis with 5VWF kit was accredited in the North Estonia Medical Centre laboratory according to ISO15189:2012. Recently, a new Hydragel 11VWF multimers assay kit (H11VW), which allows more significant sample size determinations, has become commercially available.

The aim of this study was to evaluate the performance characteristics of H11VW.

## Materials and Methods

### Study objects

Two types of normal citrated plasma samples were used in the H11VW performance evaluation: in-house reference plasma (IRP) and plasma from 10 healthy individuals recruited voluntarily. IRP has been used in North Estonia Medical Centre laboratory for a couple of years, and detailed procedure on the preparation of IRP was published previously elsewhere [11]. For healthy individuals, a well-structured questionnaire was used to obtain information about age, gender, individual/family bleeding history, medication. Information provided enabled us to classify them preliminary as non VWD individuals. Venous blood samples were collected into 3.8% NC Buffered Citrate (Vacutest KIMA s.r.l., Arzergrande, Italy) tubes, which were centrifuged at 1500 g for 15 minutes at room temperature to generate platelet-free plasma (residual platelet count < 10×10^9^/L), aliquoted and stored frozen at -70 °C until further analysis. Aliquots were thawed in a water bath (+37 °C) for 5 minutes and mixed well before testing. All participating volunteers gave their informed consent. The study was performed according to the Declaration of Helsinki and was approved by the national ethics committee.

### VWF profile (first-line tests) in healthy individuals

The VWF antigen (VWF:Ag, Liatest-VWF:Ag, Diagnostica Stago, France), factor VIII coagulant activity (FVIII:C) by a one-stage, clot-based assay (STA-ImmunoDef VIII, STA-C.K.Prest, Diagnostica Stago, France) and VWF activity [13] measured as VWF binding to the glycoprotein Ib (GPIb) receptor on the platelet surface (Innovance® VWF:Ac, Siemens Healthcare Diagnostics, ISTH nomenclature VWF:GPIbM) were measured on STA-R Evolution analyser (Diagnostica Stago, France) using commercial kits.

### VWF:MM

VWF multimers evaluation was performed on Hydrasys 2 Scan instrument (Sebia, Lisses, France), using 2.0% SDS agarose gel, direct immunofixation, visualisation with peroxidase-labelled antibody and followed by densitometry, according to manufacturer recommendations. VWF multimers were classified as low, intermediate and high molecular weight multimers (LMWM, IMWM and HMWM respectively). Densitometry data was obtained using the Phoresis software originating from Sebia. Principle of VWF:MM methodology for H5WV and separation of multimers fractions was previously described in detail [9] [10] [11] [12]. Technical steps for both kits (H5WV and H11WV) are very similar. The important differences are the metal weight holding mechanism for the blotting steps (1.8 kg for H5WV and 2.3 kg for H11WV) and a number of sample positions (5 tracks gel for H5WV and 11 tracks gel for H11WV).

### Analytical performance characteristics

We have chosen the following analytical performance characteristics of H11VW to analyse: repeatability (intra-assay variability, in gel between track variation), reproducibility (inter-assay variability, between gel variation), sensitivity, EQA performance and differences between two commercially available VWF:MM kits (H5VW and H11VW). For repeatability analysis, 11 measurements were done, each for a single non VWD volunteer individual's plasma, which was applied to 11 tracks of the gel. Consequently, intra-assay coefficient of variation (CV)% was calculated. For reproducibility analysis, VWF:MM results of the same IRP from 55 different gels runs were collected, and inter-assay CVa% was calculated. For sensitivity analysis, the single volunteer's plasma with respectively known VWF antigen value was diluted in series (1:2, 1:4, 1:6; 1:8; 1:16 and 1:32). For of the dilution series VWF:MM assay was performed on single H11VW gel together with IRP for comparison reasons. The external quality assessment (EQA) program for VWF:MM was issued by the ECAT (External quality Control of diagnostic Assays and Tests with a focus on Thrombosis and Haemostasis). In total, North Estonia Medical Centre laboratory participated in 6 cycles of EQA using H11VW. Finally, applying the results of previously performed H5VW kit performance verification (n=26) [11], corresponding results of IRP on H11VW (n=29) were collected, and statistical comparison was carried out.

### Statistical analysis

Results were reported as the mean% of the respective molecular weight fraction of multimers ± standard deviation (SD) and the coefficient of variation (CV, %). The difference between the two commercial kits (H5WV versus H11VW) was evaluated by nonparametric Man-Whitney U test on the IBM SPSS software, version 21.0 (Chicago, IL, USA). Values were considered statistically significant at p<0.05.

## Results

### Intra-assay and Inter-assay variability

**Figure 1:**
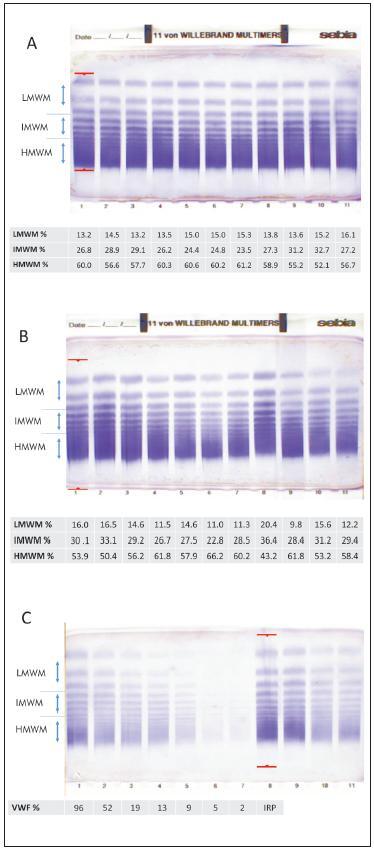
Examples of analysed H11VW gels and their semiquantitative results of VWF:MM band patterns of LMWM, IMWM and HMWM: A – repeatability analysis of a single healthy individual’s plasma in one gel (tracks 1–11); B – 10 healthy individuals’ plasmas each on a separate track (tracks 2–11), IRP (track 1); C – sensitivity analysis by serial dilution of a single healthy individual’s plasma (tracks 1–7), IRP (track 8), not relevant to the study samples (tracks 9–11). VWF, von Willebrand factor; VWF:MM, von Willebrand factor multimer assay; LMWM, low molecular weight multimers; IMWM, intermediate molecular weight multimers; HMWM, high molecular weight multimers; IRP, in-house reference plasma; H11VW, Hydragel 11VWF multimer assay kit.

Example of repeatability analysis is depicted in Figure 1A. Visually, 11 tracks of one single plasma on the same H11VW gel look pretty much the same, but a visual inspection is too subjective. Densitometric analysis of the gels, and consequently calculated % of different fractions of multimers (LMWM mean value 14.4%, SD 1.0; IMWM mean value 27.5%, SD 2.8; and HMWM mean value 58.1%, SD 2.8) demonstrate intra-assay variability performance equivalent to previously published H5VW kit repeatability values [12]: CV were 6.9% for LMWM, 10.3% for IMWM, and 4.8% for HMWM.

Statistical data in reproducibility analysis for different multimers fractions were as follows: LMWM mean value 18.1%, SD 3.0; IMWM mean value 33.1%, SD 2.0; HMWM mean value 48.9%, SD 3.9. These results yielded higher but more or less acceptable coefficients of variation for LMWH and HMWM when compared to repeatability data, but the variability of IMWM was lower. Inter-assay CV values were 16.6% for LMWM, 6.2% for IMWM, and 8.1% for HMWM, respectively.

### Healthy individuals' results

All 10 healthy individuals' plasma samples demonstrated normal FVIII:C, VWF activity and antigen levels with normal activity to antigen ratio (>0.7): FVIII:C mean value 108% (range 69-134%), VWF antigen mean value 96% (range 65-141%) and VWF activity mean value 105% (range 78-154%). Also, normal multimer patterns were detected, which resembled the normal pattern of IRP (Figure 1B). The means (ranges) for VWF:MM of different sizes were as follows: LMWM mean value 13.8% (9.8-20.4%), IMWM mean value 29.3% (22.8-36.4%) and HMWM mean value 56.9% (43.2-66.2%).

### Sensitivity analysis

Sensitivity analysis with serial dilutions revealed a cut-off (VWF antigen values 9% and below), which aggravates visual inspection of gels, worsens densitometric analysis by Phoresis software (Sebia, France). An example of sensitivity analysis is given in Figure 1C.

Plasma of a healthy individual (VWF antigen of 96% and VWF activity of 105%) was diluted according to protocol and resulted in final VWF antigen values of 52%, 19%, 13%, 9%, 5%, and 2%. As shown in Figure 1C, at the level of 9% multimer, bands are still clearly recognisable, densitometric distribution of different fractions is substantially lower when compared to IRP graph, but proportions of LMWM, IMWM and HMWM values resembled the normal values.

### EQA survey

The performance of the North Estonia Medical Centre laboratory in the ECAT Foundation EQA programs was considered successful because results of VWF:MM of all 6 cycles were in agreement with the corresponding goals of the ECAT Foundation. Summary of EQA results is provided in Table 1.

**Table 1:** Summary of VWF:MM analysis in EQA samples, reproduced with permission from the ECAT Foundation (the Netherlands)

EQA survey Nr.	EQA sample	Quantitative results, %			Interpretation	Conclusion on VWD type
		LMWM	IMWM	HMWM		
2018-M3	Type 2 VWD plasma	37.1	26.1	36.8	Relative decrease of HMWM	Type 2 VWD
2018-M4	Type 1 VWD plasma	25.0	32.3	42.5	Normal distribution	Type 1 VWD
2019-M1	Normal Coagulation Control Plasma	19.8	37.6	42.6	Normal distribution	Not VWD
2019-M2	Type 1 VWD plasma	18.0	36.5	45.5	Normal distribution	Type 1 VWD
2019-M3	Type 1 VWD plasma	19.1	28.0	52.9	Normal distribution	Type 1 VWD
2019-M4	Type 2 VWD plasma	36.5	31.0	32.5	Relative decrease of HMWM	Type 2 VWD

### Comparison between the two commercial kits, H5WV versus H11VW

**Figure 2:**
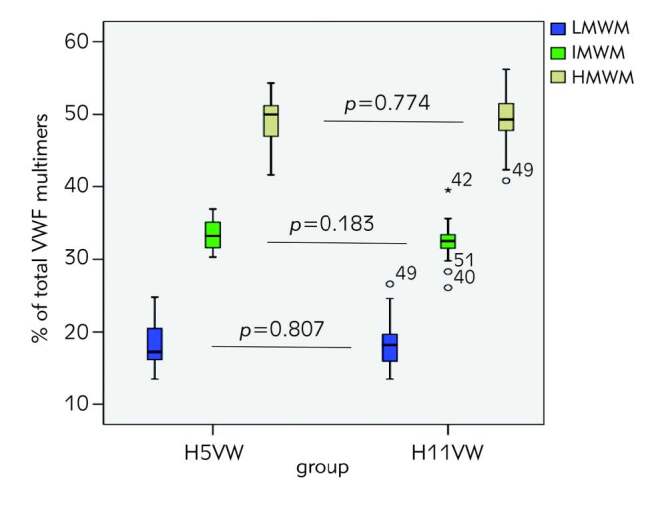
The difference between the two commercial kits (H5WV versus H11VW) represented as box plot results. LMWM, IMWM and HMWM bands are separated VWF, von Willebrand factor; LMWM, low molecular weight multimers; IMWM, intermediate molecular weight multimers; HMWM, high molecular weight multimers; H5VW, Hydragel 5VWF multimer assay kit; H11VW, Hydragel 11VWF multimer assay kit.

There was no statistically significant difference detected between H5VW and H11VW kits for different fractions of multimers: LMWM 17.95±2.94 vs 18.31±3.32, p=0.807; IMWM 33.24±1.98 vs 32.47±2.48, p=0.183; HMWM 48.82±3.65 vs 49.22±3.57, p=0.774 (Figure 2).

Following the success of the EQA and above provided performance data, in May 2020 VWF:MM assay with H11VW kit was accredited in the North Estonia Medical Centre laboratory according to ISO15189:2012.

## Discussion

The measurement of VWF multimers has become a part of the laboratory workflow for the identification and classification of VWD [1] [4]. Several home-made methods have been developed in the past decades for evaluating the VWF multimeric structure in expert-level laboratories, characterised by varying analytical performances [5] [6] [7], occasional differences in interpreting the results [12].

Both visual and densitometry-based investigation makes interpretation easier, allows the overlay patients curves with normal control and enables estimating the relative quantification of each multimer subset, providing useful information for the diagnosis of VWD. Implementation of VWF multimers assay for routine use is important for the classification of VWD, leading to the improvement of VWD diagnosis and monitoring of treatment response in Baltic countries.

The role of quality assurance in a hemostasis laboratory is very important [14]. Because high precision is needed in VWF multimers quantification, standardisation of its measurements is crucial for an accu-rate diagnosis. One important aspect is the type of plasma sample used for internal quality control (IQC) [14]. It is known that present type 2A VWD-like controls are not provided by VWF test manufacturers; thus, laboratories may be able to use previously diagnosed 2A VWD patients' samples. Using normal commercial plasma for IQC multimeric evaluation might end up with a relative loss of the highest HMWM, probably due to the lyophilisation process while preparing commercial plasma [6] [11]. To our knowledge, at least one group of researchers have verified commercial normal reference plasma (Standard Human Plasma, Siemens) as acceptable quality control for VWF multimers evaluation [15]. This study was the first to report the H11VW kit validation results, including analysis of the VWD patients' samples, and has also noted the benefits and limitations of semi-automated VWF:MM assay, including the smaller sample size H5VW kit. In the present study, the results do not only support the previously published [15] but also provide additional analytical performance characteristics evaluation, especially for the larger sample size H11VW kit.

We concluded that the analytical performance of HYDRAGEL 11 VON WILLEBRAND MULTIMERS assay is acceptable and gives a perspective to standardisation of the VWF:MM assay by Sebia (France). New H11VW kit conveniently offers a larger number of samples on a single gel, thus saves precious time. The choice of kit (H5VWvs H11VW) can be generally based on the volume of laboratory workload (the number of collected patient samples). Considering the performance data, H5VW and H11VW kits can be used in daily practice for the visual investigation of gel and quantitative estimation of VWF multimer fractions interchangeably.

## Acknowledgements

Research funding: Sebia (Lisses, France) donated the Hydragel 5von Willebrand multimers kits. The authors would like to express gratitude to dr. Lenna Örd for language editing and laboratory technicians Galina Trofimova and Tatjana Tverskaja for excellent technical assistance related to sample testing.

## Conflict of interest statement

All the authors declare that they have no conflict of interest in this work.

## List of abbreviations

VWF, von Willebrand factor; VWD, von Willebrand disease; VWF:MM, von Willebrand factor multimer assay; LMWM, low molecular weight multimers; IMWM, intermediate molecular weight multimers; HMWM, high molecular weight multimers; IRP, in-house reference plasma; H5VW, Hydragel 5VWF multimer assay kit; H11VW, Hydragel 11VWF multimer assay kit.
